# Gas Plasma Technology and Immunogenic Cell Death: Implications for Chordoma Treatment

**DOI:** 10.3390/cancers17040681

**Published:** 2025-02-18

**Authors:** Sander Bekeschus, Karl Roessler, Oliver Kepp, Eric Freund

**Affiliations:** 1 ZIK plasmatis, Leibniz Institute for Plasma Science and Technology (INP), 17489 Greifswald, Germany; 2 Department of Dermatology and Venerology, Rostock University Medical Center, 18057 Rostock, Germany; 3 Department of Neurosurgery, Medical University of Vienna, 1090 Vienna, Austria; 4 Centre de Recherche des Cordeliers, Equipe Labellisée par la Ligue Contre le Cancer, Université de Paris, Sorbonne Université, Inserm U1138, Institut Universitaire de France, 75005 Paris, France; 5 Metabolomics and Cell Biology Platforms, Institut Gustave Roussy, 94800 Villejuif, France; 6 Faculté de Médecine, Université de Paris Saclay, 94270 Kremlin Bicêtre, France

**Keywords:** brain cancer, CAP, kINPen, LTP, neurosurgery, NTP, plasma medicine, reactive oxygen species, ROS, oxidative stress

## Abstract

Cancer is the second-leading cause of death in developed societies. Specifically, cancers of the spine and brain come with significant therapeutic challenges. Chordomas are semi-malignant tumors that develop from embryonic residuals at the skull base (clival) or coccyx (sacral). Small tumor fragments can remain in the operation cavities during surgical resection, forming new tumor sites. This requires repeated surgeries or the application of proton-beam radiation and chemotherapy, which often do not lead to complete remission of the tumors. Hence, there is a need for novel therapeutic avenues that are not limited to killing visible tumors but can be applied after surgery to decrease chordoma recurrences. Reactive oxygen species (ROS) generated locally via novel medical gas plasma technologies are one potential approach to address this clinical problem. Previously, broad-spectrum free radicals generated by these cold physical plasmas operated at about body temperature were shown to oxidize cancer cells to the disadvantage of their growth and induce immunogenic cancer cell death (ICD), ultimately promoting anticancer immunity. This review outlines the clinical challenges of chordoma therapy, how medical gas plasma technology could serve as an adjuvant treatment modality, and potential immune-related mechanisms of action that could extend the longevity of gas plasma therapy beyond its acute local tissue effects.

## Simple Summary

Chordoma is a cancer of the remnant of the primitive notochord that can occur along the whole neuroaxis. Its therapy is challenging and not always successful. In recent years, new local treatments have emerged, and the importance of the immune system in attacking cancer has been shown in many tumor types.

## 1. Chordoma

**Figure 1:**
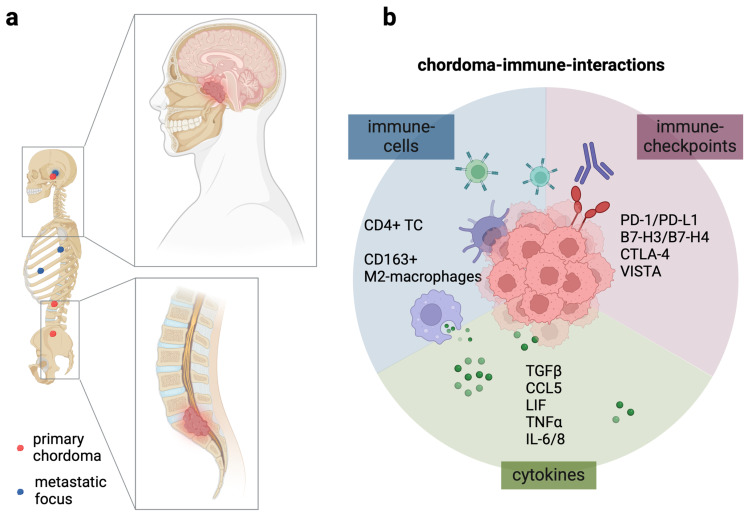
(**a**) Sites of chordoma occurrence and metastatic foci as well as local compression of tissue of the neuronal axis. (**b**) Immuno-oncological dimension of chordomas characterized by elevated M2-macrophages and T-cells, as well as expression of immune checkpoints and cytokine secretion. Figure adapted from [5].

Chordomas are rare, slow-growing tumors that arise from the notochord remnants. This structure plays a key role in early embryonic development and is instrumental in forming the spine and central nervous system. These tumors are most commonly found in the spine, particularly in the sacrum (the lower back area) and the base of the skull (clivus). Chordomas can vary in histological characteristics and are typically classified into two main types. Conventional chordoma is the most common type, characterized by a mix of chordoid and myxoid features. By contrast, dedifferentiated chordomas are more aggressive and tend to have a worse prognosis. Symptoms associated with chordomas depend on their size and location, and include pain, neurological deficits, and other symptoms related to pressure on adjacent structures, such as the spinal cord or cranial nerves. Their rarity and specific demographic patterns characterize the epidemiology of chordomas. They are estimated to have an incidence of about 0.08 to 0.2 cases per 100,000 individuals per year. Chordomas can occur at any age but are most commonly diagnosed in adults, typically between ages 30 and 70. There is a slight male predominance, with studies suggesting that chordomas occur more frequently in males than females, though the exact ratio can vary in different studies. Some studies have suggested geographic differences in the incidence of chordomas, although more research is needed to clarify this aspect. In rare cases, familial syndromes associated with chordomas have been observed, linking certain genetic predispositions, although most chordomas occur sporadically without any known hereditary component. The typical treatment option for chordomas is surgery, being the primary treatment method to remove as much of the tumor as possible. However, complete removal can be challenging due to the tumors’ proximity to critical structures. Another treatment option is radiation therapy, which may be conducted post-surgery or as a primary treatment in cases where surgery is not feasible. In addition, proton therapy and stereotactic radiosurgery are often employed due to their high precision [1]. Pharmacological intervention includes targeted therapies [2], especially if the tumor has specific genetic mutations that can be addressed, such as the EGFR/VEGFR pathway [3] and PARP inhibitors [4]. Immunotherapy is the latest addition to the chordoma treatment spectrum (Figure 1) [5]. It has been unraveled that the tumor microenvironment (TME) contains macrophages skewed toward the tumor-promoting type (TAM) and T-cells, with the latter being potential targets for, e.g., Nivolumab and Ipilimumab antibody therapy targeting the T-cell immune checkpoints PD-1 and CTLA4. The overall prognosis of chordoma largely depends on factors such as the tumor’s location, size, whether complete surgical resection was possible, and the presence of metastases. Recurrence rates can be high, especially for those not completely resected. Due to their rarity, large-scale epidemiological studies on chordomas are limited, and ongoing research continues to investigate their etiology and clinical behavior further.

## 2. Medical Gas Plasma Technology

**Figure 2:**
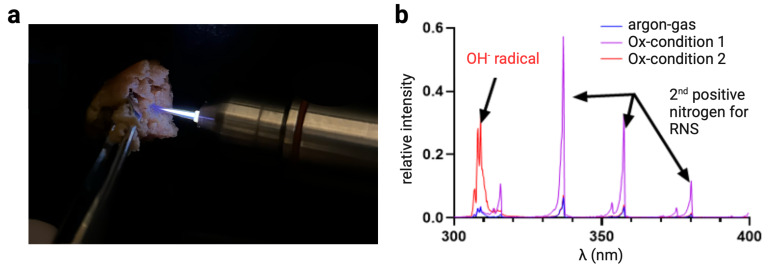
(**a**) Ex vivo tissue exposure to the atmospheric pressure medical gas plasma jet kINPen MED was used to generate various ROS types. The principle is based on argon gas being ignited at the jet head’s center by a high-frequency, high-voltage electrode; ionized argon being driven out into the ambient air; and argon ions generating radicals from ambient air oxygen and nitrogen, effectively generating reactive oxygen and nitrogen species (ROS). (**b**) Optical emission spectroscopy of the gas plasma shows distinct peaks that can be attributed to different reactive species precursors, such as hydrogel radicals (309 nm) and the second positive system of nitrogen. Figure copyright: the authors.

As opposed to blood plasma being the cell-free liquid of vertebrate blood, medical gas plasma is ionized gas, also referred to as the fourth state of matter. In the early 20th century, medical practitioners used early devices to generate plasma discharges to treat diseases [6]. After that, the medical application of gas plasmas was forgotten until the late 2000s, when, in Germany, teams of plasma physicists and medical doctors tested next-generation gas plasma devices for the treatment of chronic wounds and ulcers [7,8]. This was based on findings showing the potent antimicrobial activity of gas plasmas against bacteria and fungi [9,10], including drug-resistant strains [11,12]. Mechanistically, a significant share of this effect is mediated by a plethora of reactive oxygen and nitrogen species (ROS/RNS) generated by gas plasma devices (Figure 2) [13]. ROS/RNS are evolutionary conserved antimicrobial agents that phagocytes have used until today to inactivate engulfed microorganisms in the phagolysosome [14]. Therefore, it is unsurprising that plasma devices of various forms and operating settings have potential antimicrobial activity [15]. Another trait critical in medical applications is the relatively low temperature of modern medical gas plasma devices operated at about body temperature [13]. This allows medical gas plasma therapy to be performed without thermal harm as tissue necrosis is not induced, which is opposite to long-existing hotter gas plasma devices, such as the argon plasma coagulator, that are explicitly used to generate higher temperatures to facilitate tissue cutting during electrosurgery or wound sealing by inducing hemostasis [16].

Gas plasmas are potent ROS/RNS producers that play multiple roles in the human body. Besides being a byproduct of cellular metabolism, low levels of ROS/RNS can also serve as signaling agents critical in redox regulation and adaption, cellular differentiation, and growth stimulation [17,18]. At low gas plasma exposure times or treatment intensities, such products are also induced in, for example, chronic wounds via induction of the transcription factor Nrf2 and the Hippo pathway [19,20], ultimately promoting healing responses [13]. However, similar to pharmacological agents, the effects of ROS/RNS depend on their concentration. While having potentially stimulating properties at lower levels, high amounts of ROS/RNS are detrimental, leading to overwhelming cellular regulation and antioxidant defense systems and eventually leading to regulated or non-regulated cell death [21]. This concept is called hormesis and has also been described for approved drugs and other pharmacological agents [22,23]. Accordingly, high ROS/RNS levels can have tumor-toxic effects, a concept exploited for decades through various anticancer therapies, such as photodynamic therapy (PDT) [24], radiotherapy [25], and selected pharmacological agents [26]. Therefore, it comes as no surprise that medical gas plasma has been shown to have anticancer effects at the point of application. In screening experiments of cells of various tumor entities, it has already been demonstrated that antitumor effects arise in all models investigated, regardless of the malignant primary cell lines or specific mutations [27]. The first in vivo evidence of plasma-mediated tumor control was published in 2010 in a model of glioblastoma xenografts [28]. Since then, the principle has been successfully iterated using a range of gas plasma device systems and operating conditions and in many different cancer types in vivo, including breast cancer [29], squamous cell carcinoma [30], melanoma [31], brain tumors [32], bone cancer [33], and gynecological tumors [34]. Again, it has been shown that ROS/RNS significantly contribute to the mode of action of gas plasma-mediated cytotoxicity [35]. This complex plasma redox environment could provide an advantage over conventional radiation, which tends to generate only superoxide anions and hydroxyl anions that only diffuse intracellularly by a few millimeters. These treatment modalities also require a clear treatment target, while tumor remnants are often only clearly visible in the MRI when they are several millimeters in size, and scattered cells, which can also form metastases, remain invisible [36].

## 3. Immunogenic Cell Death (ICD)

The realization of the potent activity and contribution of the immune system to tumor control is the most remarkable step forward in oncology in the 21st century so far. While in basic and translational research, findings on anticancer immunity date back to decades ago, it was not until the Nobel Prize for antibody checkpoint immunotherapy in 2018 that the fields of tumor immunology and cancer immunotherapy gained global recognition [37]. For cancer immunotherapy to work, for example, by utilizing antibodies blocking immunosuppressive molecules on T-cells to unleash their full antitumor potential, which is otherwise blocked by cancer surface molecules [38], pre-existing T-cell clones targeted against tumor-associated antigens (TAA) or neo-antigens are required [39,40]. This is feasible because tumor cells have acquired genetic mutations throughout their evolution to evade growth-inhibiting and immune control [41]. At the same time, the DNA repair machinery is often dysfunctional, leading to a high mutation burden, greater genetic variety, adaptability, and altered peptide sequences being presented on the cancer cell’s surface. T-cells harboring the respective T-cell receptor can detect and identify these as foreign, potentially allowing their killing. This, however, requires that the tumor antigens have been presented to T-cells before in an inflammatory context to signal the potential danger of cells displaying such antigens. Notably, checkpoint immunotherapy gained importance in chordoma therapy [42,43,44,45,46], and T-cell responses can predict the outcome of the disease [47]. Every successful immunotherapy has shown that immune attack can be universal, which suggests that atypical tumors such as chordoma can also benefit from it. In particular, processes such as the induction of ICD can play a decisive role in the future of this tumor.

The classical representation of cell death distinguishes between a regulated form, where apoptosis is activated via an extrinsic or intrinsic signaling pathway, and free and accidental dying due to damage to cell structures, i.e., necrosis [48,49]. In necrosis, cellular components are released into the surroundings, whereas in apoptosis, cellular remnants are packaged into vesicles and cleared away [50]. This concept is constantly being expanded. For example, individual signaling cascades or functional consequences can be associated with cell death forms such as ferroptosis, pyroptosis, autophagy, necroptosis, or cornification [51,52]. ROS/RNS can also induce some of these in supraphysiological concentrations [53]. The importance of this regulated, or even programmed, cell death is also reflected in its associated higher energy consumption for a cell [54]. Still, in this way, it can contribute to good tissue homeostasis [49,50]. The mechanisms involved are essential, such as removing senescent cells, pathogens, or malignant cells or selecting immune cells. ICD involves the regulated secretion or membrane expression of the cell’s factors, which can contribute to the activation of immune cells [55]. These factors include heat shock proteins (HSP), high-mobility group protein B1 (HMGB1), adenosine triphosphate (ATP), and calreticulin (CRT) [55,56]. By binding to innate immune system receptors (pathogen-recognition receptors, PRRs), defense mechanisms are initiated that are deeply rooted in immune memory [57]. The factors mentioned thus act as danger signals (damage-associated molecular patterns, DAMPs) [56]. In the context of tumor cell death, antigen-presenting cells can be activated, and the phagocytosis of tumor cells can be initiated [55,56,58,59]. The resulting stimulation of adaptive immune cells can lead to various reactions in the context of DAMPs and an antitumor immune response. ICD can provide such a danger-context [60]. For example, various chemotherapeutic agents, ionizing radiation, photodynamic therapy, infections, and high hydrostatic pressures can contribute to initiating ICD with varying degrees of effectiveness [55]. The expression of the chaperone of the endoplasmic reticulum (ER), calreticulin, on the cell membrane of dying tumor cells is particularly indicative of this [61]. Obeid et al. were able to show in vitro that several established chemotherapeutic agents had different potentials for the membrane expression of CRT when inducing the same classical cell death features, such as the membrane expression of phosphatidylserine or caspase activation [51,61]. Subsequently, the tumor cells were inactivated using different methods and used as a vaccine in an animal model. The chemotherapeutic agents that led to a strong membrane expression of CRT and thus had an immunogenic effect were highly effective when injected with vital tumor cells [61]. Addressing such an immunogenic effect, apart from the toxic component of tumor therapy, is particularly relevant in combination regimens and could thus become more important in the clinic [62].

## 4. Gas Plasma-Induced ICD to Improve Anti-Chordoma Immunity

ICD is a form of cell death that not only results in the elimination of cells but also stimulates and activates immune responses. This process is characterized by specific changes in the dying cells that signal to the immune system, promoting the recognition and killing of other similar cells that may be malignant or infected. Key features of ICD include DAMP release to alert immune cells to the presence of dying or damaged cells, phagocytosis being promoted by DAMPs and critical for proper antigen processing, T-cell activation via above-threshold antigen presentation by DCs, and cytokine release that can further enhance the immune response. Medical gas plasma technology could kick-start this process by ICD induction to add to its tumor-toxic effects at higher intensities. Such immune responses are not limited to visible tumor residues (as explanted by surgeons) but can tackle micro-residual cancer cells that cause chordoma spread and recurrence [63,64]. Cancers are often infiltrated by immune cells, but many types of tumors hijack immune cells and their functions to favor tumor growth via, e.g., tumor-associated macrophages (TAM) or tumor-associated neutrophils (TAN) [65,66]. Here, the local gas plasma treatment of chordoma could also contribute to a shift of phenotypes in the TME. In addition, DCs potentially could increase their activity in gas plasma-treated chordoma tumor tissue. In the TME, DCs always take up tumor antigens but often do so in a non-inflammatory context, promoting immunosuppression rather than providing the co-stimulation necessary to unleash antitumor T-cell responses. Activating professional antigen-presenting cells such as DCs in the chordoma TME could be critical in tackling chordoma.

**Figure 3:**
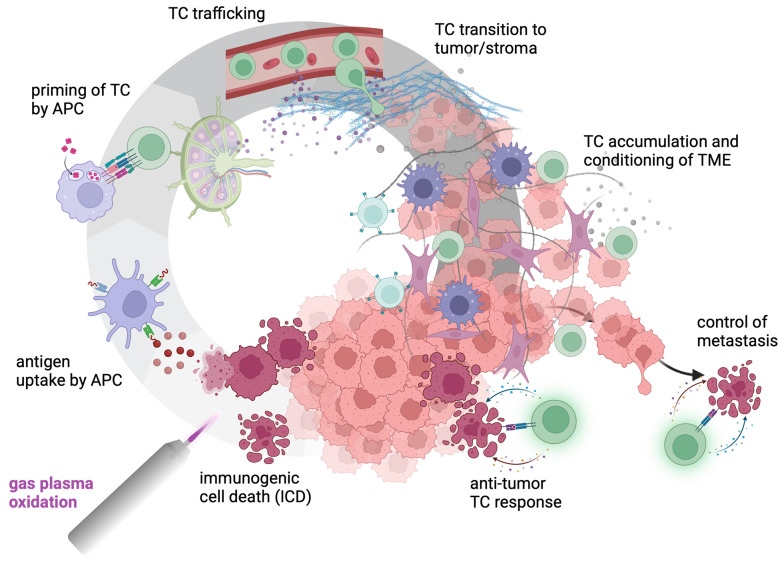
Principles of immunogenic cell death (ICD), modified after [81]. Therapy-induced stress, such as through the local delivery of high levels of ROS via medical gas plasma technology, induced an inflammatory type of cell death in chordoma cells, characterized by DAMP release (e.g., ATP) and exposure of pro-phagocytic signals (e.g., calreticulin, CRT), leading to enhanced phagocytosis by antigen-presenting cells (APCs). Activated by DAMPs and ICD, APC maturation and migration into the draining lymph node lead to improved tumor antigen presentation, e.g., to T-cells (TCs), rendering them into effector cells entering the circulation to target tumor cells systemically and condition the tumor microenvironment (TME).

Gas plasma and its derived ROS/RNS were previously shown to induce ICD in several tumor entities. Studies showed that ICD is induced independently of toxicity and only by a minor fraction of chemotherapeutics [61]. The hypothesis was that exogenous gas plasma-derived ROS/RNS could shape the TME, cell viability, and metabolism, leading to the DAMPs (Figure 3). ROS/RNS and potentially other gas plasma parameters could induce stress in cancer cells, leading to ER stress and CRT translocation, DAMP release, and molecules associated with inflammatory cell death. This was shown for the first time for two different gas plasma device principles, DBDs and plasma jets, in 2017 [67,68,69]. Compared to DBDs, which have a flat geometry [70], gas plasma jets are particularly useful for spot-like treatments and when reaching cavities is required [71]. As outlined in the next section, this would also be necessary for the chordoma treatment. Gas plasma jets produce various ROS/RNS and deposit these into the gas phase, liquids, and tissues. These ROS/RNS and oxidants are associated with changes in the TME and tumor-infiltrating leukocytes (TILs) in syngeneic animal tumor models in vivo (Table 1). For instance, it was previously found that the repeated injection of plasma-treated liquid into the peritoneal cavity of tumor-bearing mice strongly reduced pancreatic and colorectal cancer growth [72,73]. In both models, elevated numbers of macrophages harboring fewer features of tumor-promoting M2 phenotypes were observed, as well as markers of inflammation-associated cell death [74]. Using the direct gas plasma treatment of subcutaneous tumors in syngeneic cancer models revealed significantly increased numbers of intratumoral CD4^+^ and CD8^+^ T-cells and DCs, which moreover were dependent on the type of ROS/RNS mixture generated by medical gas plasma technology, suggesting a direct relationship between ROS/RNS, antitumor effects, and changes in TIL composition and activity [75]. Intriguingly, such processes were exacerbated when gas plasma therapy was combined with immune checkpoint antibody therapy [76]. All these effects were achieved with the kINPen, a medical product from Germany approved for clinical applications [77]. Similar findings were also made with other, not-approved, experimental gas plasma jet devices, especially regarding inducing abscopal effects in mice [78,79,80]. These studies suggest that gas plasma jet devices are suited not only to kill tumor cells but also to induce inflammatory types of cell death that are associated with an altered TME and TIL composition, potentially adding antitumor immunity-induced cancer cell killing to the initial toxicity elicited by gas plasma-generated ROS/RNS.

## 5. Bridging Laboratory and Clinical Discovery

**Figure 4:**
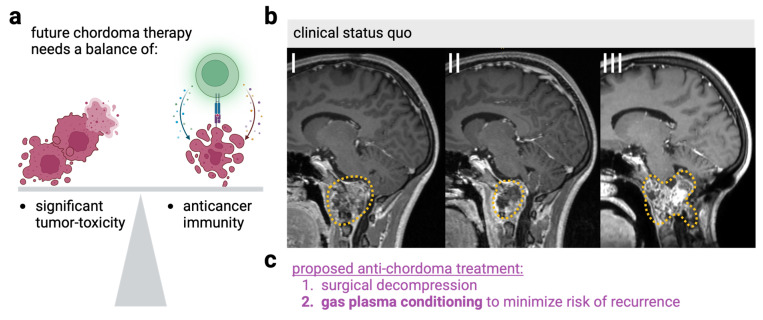
(**a**) Sketch of the two aspects of direct tumor toxicity and anticancer immunity that are needed to balance future effective chordoma therapies. (**b**) MRI images (T1-weighted with contrast medium) show the typical clinical course of an aggressive chordoma (**I**) at time of diagnosis, (**II**) after surgical decompression of the brain stem and important neuronal structures, and (**III**) after its recurrence as extensive disease. (**c**) Summary of the proposed chordoma treatment that adds gas plasma oxidation after surgical decompression to minimize the risk of tumor recurrence. Figure copyright is by the authors.

Despite the low grade of malignancy, therapy of chordoma remains challenging [84]. Even radical surgery without signs of macroscopic residuals often leads to tumors re-emerging in the course of months and years post-surgery. The vision of utilizing medical gas plasma technology to support clinical chordoma therapy has three dimensions. The first is the induction of primary cell death in gas plasma-treated tissue. The second relates to ICD elicitation and subsequent systemic immune-mediated cancer control. The third is about how to design gas plasma devices efficiently for use during surgery (Figure 4).

Medical gas plasma device exposure is remarkably well-tolerated [13], which is particularly important in the brain for numerous critical structures, such as the brain stem or cranial nerves. The low temperature of the treatment, minimal mechanical manipulation, and the absence of electrical currents are particularly advantageous. At the same time, because of its relatively mild character, it is less suitable for tumor debulking, for in the case of chordoma, surgical removal remains the primary choice of therapy. However, surgical chordoma removal is often incomplete, leaving microtumors behind the tumor resection margins, thus giving rise to new chordoma tissue. Here, medical gas plasma technology could be used to treat the tumor resection margins, eradicating tumor cells remaining in such tissues. This could ultimately reduce chordoma recurrences and, therefore, additional surgery, radiation, or proton-beam therapy. Plasma jet systems are already capable of precise, scalpel-like treatment that can be applied focally by the surgeon’s hand. By carefully guiding the device, individual spots can be treated, for example, by exposing certain tumor residues to oxidation for a longer period of time. The working channel on the other hand can also be treated wider and shorter if only individual cells are suspected. Hemostasis is an essential point in any surgical treatment. In such a treatment of the working channel, it could also be helpful that even short gas plasma exposure leads to strong platelet activation and, thus, to gentle coagulation compared to electrocautery [85]. While ROS/RNS are generally thought to have a limited penetration ability into tissues, dozens of studies on mouse models harboring subcutaneous tumors of different types have previously suggested gas plasma-derived ROS/RNS to reach the tumor, ultimately leading to tumor reduction [86]. We have also shown that tumor cells can be inactivated by gas plasma within hydrogels [35], further suggesting the deep effects of this partially ionized gas into tissue. In addition, in palliative patients, gas plasma exposure to oral squamous cell carcinoma succeeded in several patients in reducing local tumor burden and extending expected survival [87]. Moreover, we have shown in five tumor entities, including glioblastoma multiforme, that exposure to gas plasma ex vivo readily induced apoptosis in tumor tissues [88,89,90]. This indicates similar findings could be made in chordoma tissue ex vivo and in vivo. Given the previous findings on ICD with gas plasma treatment, it is also conceivable that local gas plasma exposure could induce ICD in chordoma. By mounting anti-chordoma immunity, chordoma-targeting T-cells could attack chordoma recurrences in locations other than the location of the primary gas plasma exposure or keep microsatellites from forming larger tumor masses. This could be achieved using small-diameter microplasma jet devices—small enough to fit into an endoscopic channel—as these are currently being developed [91,92]. During the microsurgical removal of chordoma, the procedure involuntarily transports tumor tissue into the cavity used for the insertion of surgical equipment. Immediately afterward, an endoscope of the same size with an angle gas plasma jet could be used to gradually expose this cavity to ROS/RNS, which could inactivate superficial tumor cells or, closer to the original tumor site, microsatellite tumors present in the tumor resection margins. The principle validity of our approach has been shown in a small cohort of chordoma patients receiving vaccination with heat-killed chordoma in a therapeutic context [43], as heat-killing is known to generate ROS endogenously [93]. The idea that ROS play a critical role in enhancing immunogenicity was recently tested in ovarian cancer patients receiving therapeutic vaccination with ROS-killed autologous tumor material. Several patients showed enhanced T-cell responses and improved therapeutic outcomes [94]. Therefore, we believe that medical gas plasma technology ideally combines both research lines to benefit chordoma patients by amplifying antitumor immunity in the future.

## 6. Conclusions

Chordoma is a tumor with high recurrence, especially within the microsurgical channel employed for tumor removal. Future studies and therapeutic concepts could explore the opportunities of medical gas plasma technology, characterized by its high-level production of various reactive species types and capability of inducing ICD and mounting new or enhancing existing anticancer immunity. As an intrasurgical tool to treat the microchannels after chordoma removal, gas plasma could be a disruptive technology to reduce chordoma recurrences in patients in the future.

## Figures and Tables

**Table 1:** In vivo studies of syngeneic mouse models using cold gas plasma jets operated at atmospheric pressure and suggesting immune-oncology effects of cold physical plasma exposure.

First Author	Mouse and Tumor Model	Plasma Jet and Gas Type	Main Findings	Ref.
Freund et al.	Balb/c and CT26 (colorectal cancer)	kINPen jet, argon, plasma-treated liquid	increased macrophages and T-cell activation	[73]
Chen et al.	C57BL/6 and B16F10 (melanoma) and Balb/c and 4T1 (breast cancer)	prototype plasma patch needle array	increased TIL and combined effect with PD-1 checkpoint immunotherapy	[82]
Liedtke et al.	C57BL/6 and PDA6606 (pancreatic cancer)	kINpen jet, argon, plasma-treated liquid	increased macrophages but decreased M2 types	[83]
Miebach et al.	Balb/c and CT26 (colorectal cancer)	kINPen jet, argon, plasma-treated liquid	enhanced ICD	[74]
Mahdikia et al.	Balb/c and 4T1 (breast cancer)	prototype helium plasma jet	abscopal effects, increased T_H_17 TILs	[80]
Bekeschus et al.	C57BL/6 and B16F10 (melanoma)	kINPen jet, argon/argon-oxygen/helium/helium-oxygen	depending on feed gas type used, increased T-cells and dendritic cells	[75]
Miebach et al.	C57BL/6 and B16F10 (melanoma)	kINPen jet, argon	combined effect and increased TIL with PD-1 checkpoint immunotherapy	[76]
